# Netarsudil‐Induced Honeycomb Keratopathy in an Eye With Iridocorneal Endothelial Syndrome

**DOI:** 10.1155/crop/8615059

**Published:** 2026-09-18

**Authors:** Alina N. Ferguson, Karine Duarte Bojikian, Minh T. Nguyen

**Affiliations:** ^1^ Department of Ophthalmology, University of Washington, Seattle, Washington, USA, washington.edu; ^2^ University of Washington School of Medicine, Seattle, Washington, USA, washington.edu; ^3^ Casey Eye Institute, Oregon Health & Science University, Portland, Oregon, USA, ohsu.edu

**Keywords:** honeycomb keratopathy, ICE syndrome, iridocorneal endothelial syndrome, netarsudil, Rhopressa

## Abstract

**Purpose:**

To describe a case of netarsudil‐associated honeycomb keratopathy in a patient with iridocorneal endothelial (ICE) syndrome.

**Observations:**

A 43‐year‐old male with a history of Cogan–Reese variant ICE syndrome presented with ocular irritation, foreign body sensation, blurred vision, photosensitivity, and discharge in the right eye 2 weeks after initiating topical netarsudil 0.02% for secondary angle‐closure glaucoma. Slit lamp exam and anterior segment optical coherence tomography demonstrated diffuse reticular epithelial cysts, consistent with netarsudil‐induced honeycomb keratopathy. Three weeks after netarsudil cessation, slit lamp exam showed complete resolution of corneal edema.

**Conclusions and Importance:**

Endothelial cell dysfunction in ICE syndrome may be a risk factor for patients to develop reversible netarsudil‐induced honeycomb keratopathy. Ophthalmologists should exercise caution when prescribing netarsudil for glaucoma management in patients with pre‐existing corneal pathologies, including those with ICE syndrome.

## 1. Introduction

Netarsudil 0.02% ophthalmic solution (Rhopressa; Alcon, Geneva, Switzerland) is a Rho‐kinase inhibitor approved by the United States Food and Drug Administration in 2017 to reduce intraocular pressure (IOP) in patients with open‐angle glaucoma or ocular hypertension. Side effects of netarsudil included conjunctival hyperemia, conjunctival hemorrhage, corneal verticillata, and a distinct pattern of reticular epithelial edema known as honeycomb keratopathy [1, 2].

Honeycomb keratopathy has been increasingly observed in patients using netarsudil, particularly those with pre‐existing endothelial dysfunction or prior intraocular surgery such as glaucoma drainage device implantation, penetrating keratoplasty, or endothelial keratoplasty [3–10]. However, the condition can also occur in eyes without pre‐existing corneal disease [11]. The corneal epithelial cysts appear in a reticular, honeycomb‐like pattern and in most cases resolve completely upon discontinuation of netarsudil [9, 12–15].

Iridocorneal endothelial (ICE) syndrome is a rare disorder characterized by abnormalities of the iris and the corneal endothelium, which are caused by the increased proliferation and migration of corneal endothelial cells toward the iridocorneal angle and onto the iris [16]. ICE is sporadic in presentation, usually unilateral, and typically affects adult patients. It comprises a unique group of three ocular pathologies: Chandler syndrome, progressive iris atrophy, and Cogan–Reese syndrome [17–22]. ICE syndrome leads to corneal edema and decompensation, and secondary glaucoma. Glaucoma can develop by obstruction of the angle by the proliferating endothelial cells, or by contraction of the cells′ basement membrane over the iris and formation of peripheral anterior synechiae (PAS) [23]. Glaucoma tends to be more severe in Cogan–Reese syndrome and progressive iris atrophy than in Chandler syndrome, likely because of a higher prevalence of ICE cell proliferation across the iridocorneal angle and resulting secondary PAS in these variants [24, 25]. IOP is also significantly more difficult to control in patients with Cogan–Reese or progressive iris atrophy than in those with Chandler syndrome, regardless of the extent of PAS [25].

Herein, we present a patient with Cogan–Reese variant ICE syndrome who developed honeycomb keratopathy 2 weeks after starting netarsudil for the treatment of secondary angle‐closure glaucoma.

## 2. Case Presentation

A 43‐year‐old male with a history of Cogan–Reese variant ICE syndrome and secondary angle‐closure glaucoma of the right eye (OD) presented to a glaucoma clinic in the community for follow‐up. He had visual acuity (VA) of 20/20, IOP of 31 mmHg, and pachymetry of 534 *μ*m OD. Slit lamp biomicroscopy OD revealed 2 corneal bullae, beaten bronze appearance of the endothelium, corectopia, ectropion uvea, tan iris nodules, and an optic disk with cup‐to‐disk ratio of 0.5. The left eye was unremarkable. Because of high IOP despite topical dorzolamide‐timolol 2‐0.5% twice daily and brimonidine 0.15% twice daily, the patient was started on netarsudil 0.02% ophthalmic solution once daily and latanoprostene bunod 0.024% ophthalmic solution (Vyzulta; Bausch Health, Laval, Quebec, Canada) once daily to improve IOP control.

**Figure 1:**
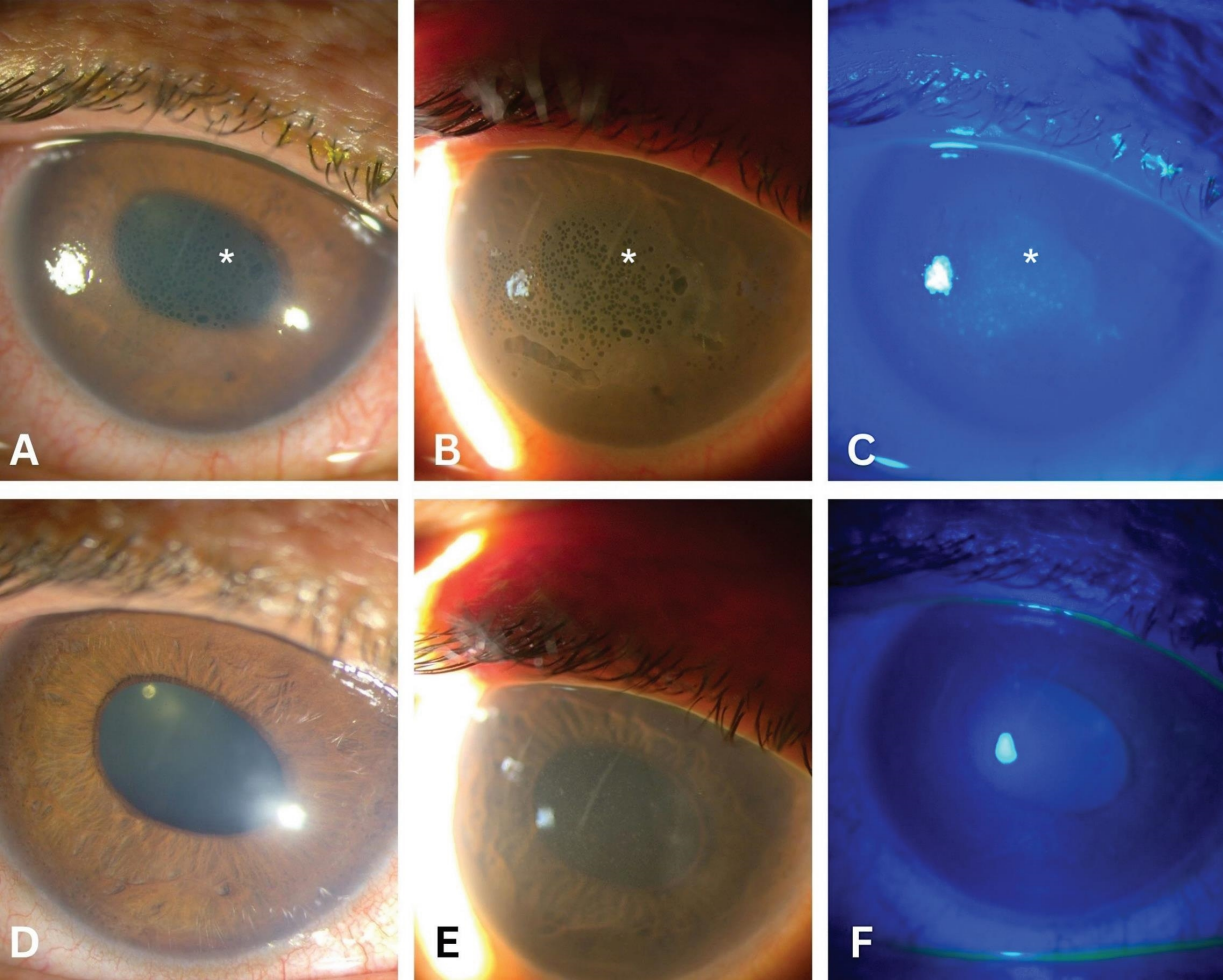
Slit lamp photography of reversible netarsudil‐induced honeycomb keratopathy in the right eye, which has features of Cogan–Reese variant ICE syndrome, such as corectopia and ectropion uvea. (Figure 1 A–C) Diffuse honeycomb‐like corneal cysts ( ^∗^) 2 weeks after starting netarsudil. (Figure 1 D–F) Resolution of honeycomb‐like corneal cysts 3 weeks after discontinuing netarsudil.

**Figure 2:**
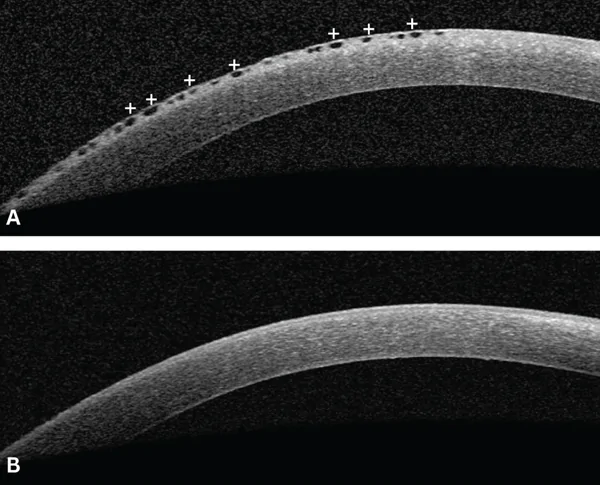
Anterior segment optical coherence tomography of the right eye. (Figure 2A) Numerous intraepithelial cysts (+) 2 weeks after starting netarsudil. (Figure 2B) Resolution of intraepithelial cysts 3 weeks after discontinuing netarsudil.

At the follow‐up appointment with his cornea specialist at the university clinic 2 weeks later, the patient endorsed foreign body sensation, blurred vision, photosensitivity, and tearing since his last glaucoma visit. VA worsened to 20/150, although IOP improved to 12 mmHg OD. Slit lamp biomicroscopy revealed central honeycomb‐like cysts with an inferior bulla (Figure 1A–C). Anterior segment optical coherence tomography (AS‐OCT) of the right eye showed numerous intraepithelial cysts that were concentrated in the inferotemporal two‐thirds of the cornea (Figure 2A). These findings were concerning for netarsudil‐induced honeycomb keratopathy, so the patient was instructed to discontinue netarsudil and latanoprostene bunod, and start acetazolamide 250 mg twice a day.

Three weeks after discontinuing netarsudil and latanoprostene bunod, VA improved to 20/80 with an IOP of 18 mmHg OD. Slit lamp exam (Figure 1D–F) and AS‐OCT (Figure 2B) revealed resolution of honeycomb‐like cysts. He underwent uncomplicated cataract surgery with intraocular lens (IOL) and Baerveldt glaucoma implant placement a week later. The IOL power was selected to leave the patient mildly myopic (−1.0 D) in anticipation of possible future endothelial keratoplasty. At 1‐month postoperative visit, his best‐corrected VA was 20/25, IOP was 18 mmHg, his cornea was clear with no cystic edema, and pachymetry improved to 493 *μ*m.

## 3. Discussion

Netarsudil‐induced honeycomb keratopathy is a well‐documented adverse effect of topical netarsudil, particularly in patients with underlying endothelial cell dysfunction. However, to our knowledge, this pattern of epithelial changes has not been previously reported in patients with ICE syndrome, a condition characterized by abnormal proliferation of corneal endothelial cells.

The mechanism by which endothelial dysfunction predisposes to reticular epithelial edema remains unclear. One theory is that improved endothelial function leads to posterior stromal clearing, shifting residual edema anteriorly into the epithelium. However, recent studies on primary human corneal endothelial and epithelial cells have revealed data that appear to refute this hypothesis [26]. Another theory proposes a direct effect of Rho‐kinase inhibitors on epithelial tight junctions and actin cytoskeleton organization, potentially contributing to epithelial bullae formation [9, 14, 26, 27]. Supporting this, animal studies with ripasudil have shown transient morphological changes in corneal endothelial cells, attributed to reduced actomyosin contractility through inhibition of focal adhesions and actin stress fibers [28, 29]. Similarly, a prospective human study observed transient ripasudil‐induced changes, such as indistinct cell borders and pseudoguttae, without evidence of endothelial cell loss [30]. Subsequent human studies further supported this theory, showing that netarsudil‐associated reticular bullous epithelial edema may result from direct, reversible transcriptional alterations in cell junction–associated genes and from indirectly increased paracellular permeability secondary to epithelial actin cytoskeleton disruption, facilitating stromal fluid entry in eyes with compromised endothelium. Gene expression normalized to control levels within 6 days of discontinuing netarsudil after 3 days of treatment, supporting the reversibility of these changes [26].

Multiple reports indicate that the netarsudil‐associated honeycomb keratopathy develops primarily in the inferior cornea [8, 9, 14]. Likewise, this case report demonstrated the greatest development in the inferior cornea. Tran et al. propose that this preferential involvement is secondary to the pooling of the medication after the administration of drops. Similarly to this case, a pattern of resolution in which the individual bullae become smaller and more widely spaced apart has been described [31]. Although most cases of honeycomb keratopathy have been completely reversible following cessation of netarsudil, there is variation in the timelines of reticular epithelial cystic edema development and resolution [9, 12, 13, 31]. Development has been described to occur within weeks or more than 6 months of starting netarsudil. Improvement has been found to occur within 1 week to 3 months after medication cessation; this case report′s resolution within 3 weeks postcessation fits this previously observed timeline [14]. Notably, there has also been an instance in which honeycomb keratopathy resolved despite the continuation of netarsudil, which was attributed to more robust healing capabilities in young patients [31].

Latanoprostene bunod is an IOP‐lowering agent with a dual mechanism of action, metabolizing into latanoprost acid, which increases uveoscleral outflow, and nitric oxide, which enhances trabecular meshwork outflow via smooth muscle relaxation. Although it was initiated and discontinued together with netarsudil in this case, latanoprostene bunod is an unlikely causative agent, as there are no reported cases of subepithelial bullae or reticular corneal changes associated with its use [32, 33]. This further supports netarsudil as the causative agent.

In conclusion, netarsudil should be used with caution in patients with underlying corneal disease, including ICE syndrome, as it may cause honeycomb keratopathy that typically resolves with discontinuation of the drug.

## Author Contributions

All authors attest that they meet the current ICMJE criteria for authorship. All authors contributed to this case report equally. Minh T. Nguyen has full access to all of the data in this study and takes complete responsibility for the integrity of the data and the accuracy of the data analysis.

## Funding

This study was supported by the Research to Prevent Blindness, 10.13039/100001818.

## Disclosure

Minh T. Nguyen affirms that this manuscript is an honest, accurate, and transparent account of the study being reported; that no important aspects of the study have been omitted; and that any discrepancies from the study as planned (and, if relevant, registered) have been explained. All authors have read and approved the final version of the manuscript.

## Consent

Per University of Washington Institutional Review Board—Human Subjects Division policy, case reports of up to three patients do not require individual patient consent.

## Conflicts of Interest

The authors declare no conflicts of interest.

## Data Availability

The data that support the findings of this study are available from the corresponding author upon reasonable request.
